# Recent advances in understanding object recognition in the human brain: deep neural networks, temporal dynamics, and context

**DOI:** 10.12688/f1000research.22296.1

**Published:** 2020-06-11

**Authors:** Susan G. Wardle, Chris I. Baker

**Affiliations:** 1 Laboratory of Brain and Cognition, National Institute of Mental Health, National Institutes of Health, Bethesda, MD, 20892, USA

**Keywords:** object recognition, human vision, fMRI, MEG, DNN, visual perception

## Abstract

Object recognition is the ability to identify an object or category based on the combination of visual features observed. It is a remarkable feat of the human brain, given that the patterns of light received by the eye associated with the properties of a given object vary widely with simple changes in viewing angle, ambient lighting, and distance. Furthermore, different exemplars of a specific object category can vary widely in visual appearance, such that successful categorization requires generalization across disparate visual features. In this review, we discuss recent advances in understanding the neural representations underlying object recognition in the human brain. We highlight three current trends in the approach towards this goal within the field of cognitive neuroscience. Firstly, we consider the influence of deep neural networks both as potential models of object vision and in how their representations relate to those in the human brain. Secondly, we review the contribution that time-series neuroimaging methods have made towards understanding the temporal dynamics of object representations beyond their spatial organization within different brain regions. Finally, we argue that an increasing emphasis on the context (both visual and task) within which object recognition occurs has led to a broader conceptualization of what constitutes an object representation for the brain. We conclude by identifying some current challenges facing the experimental pursuit of understanding object recognition and outline some emerging directions that are likely to yield new insight into this complex cognitive process.

## Introduction

Object recognition is one of the classic “problems” of vision ^1^. The underlying neural substrate in humans was revealed by classic neuropsychological studies which pointed to selective deficits in visual object recognition following lesions to specific brain regions ^2, 3^, yet we still do not understand how the brain achieves this remarkable behavior. How is it that we reliably ^4^ and rapidly ^5^ recognize objects despite considerable retinal image transformations arising from changes in viewing angle, position, image size, and lighting? Much experimental and computational work has focused on this problem of invariance ^4, 6– 13^. Early neuroimaging studies of object recognition using functional magnetic resonance imaging (fMRI) focused on regions in the lateral occipital and ventral temporal cortex, which were found to respond more strongly to the presentation of objects than to textures or scrambled objects ^14, 15^. More recently, the application of multivariate analysis techniques has led to broader investigation of the structure of object representations ^a^ throughout the ventral temporal cortex ^16, 17^ and their temporal dynamics across the whole brain ^18, 19^. While these representations are assumed to contribute to object recognition behavior, they may also contribute to other tasks. This shift toward object representations has also accompanied a greater focus on revealing how a broad range of different object categories are represented rather than investigating the invariant representation of single objects. Such object categorization involves a similar issue of extrapolation across changes in visual features as invariance, since exemplars (e.g. Great Dane and Chihuahua) of a category (e.g. “dog”) often have significantly different visual features from one another.

The aim of this review is to provide an overview of recent advances in understanding object recognition in the human brain. In this review, we primarily consider contemporary work from the past three years in human cognitive neuroscience, identifying the current trends in the field rather than providing an exhaustive summary. In addition, we focus on the neural basis of visual object recognition in the human brain (for reviews including non-human primate studies, see 20, 21) rather than the related topics of computer vision, object memory, and semantic object knowledge. We define visual objects as meaningful conjunctions of visual features ^13^ and object recognition as the ability to distinguish an object identity or category from all other objects ^21^. Face recognition is not covered in this review, as faces are a unique object class that are processed within a specialized network of regions ^22, 23^.

We identify three current trends in the approach towards understanding object recognition within the field of cognitive neuroscience. Firstly, the rapidly growing popularity of deep neural networks (DNNs) has influenced both the type of analytic approach used and the framework from which the questions are asked. Secondly, the adaptation of multivariate methods to time-series neuroimaging methods such as magnetoencephalography (MEG) and electroencephalography (EEG) has highlighted the importance of considering the temporal dynamics in the neural processing of object recognition at a resolution not accessible with fMRI. Finally, the field has begun to move away from examining single objects in isolation towards examining objects within more naturalistic contexts including a variety of both task and visual contexts. In the sections below, we examine each of these trends in turn.

## Deep neural networks as models of object vision

DNNs are a class of brain-inspired computer vision algorithms ^24– 26^. Although there are many variants of the specific network architecture, the term DNN refers to artificial neural networks in which there are multiple (i.e. “deep”) layers in-between the input and output stages ^27^. DNNs have risen to prominence within cognitive neuroscience relatively recently given high levels of performance in object classification ^28^, in some cases even performing as well as humans ^29^. This has led to consideration of the utility of DNNs as potential models of biological vision ^26, 30^. However, overall performance does not necessarily indicate that the underlying processing is similar to that in the brain. In this section, we highlight several fundamental differences between state-of-the-art DNNs and the brain and consider the potential of DNNs to inform our understanding of human object recognition given these differences.

**Figure 1.**
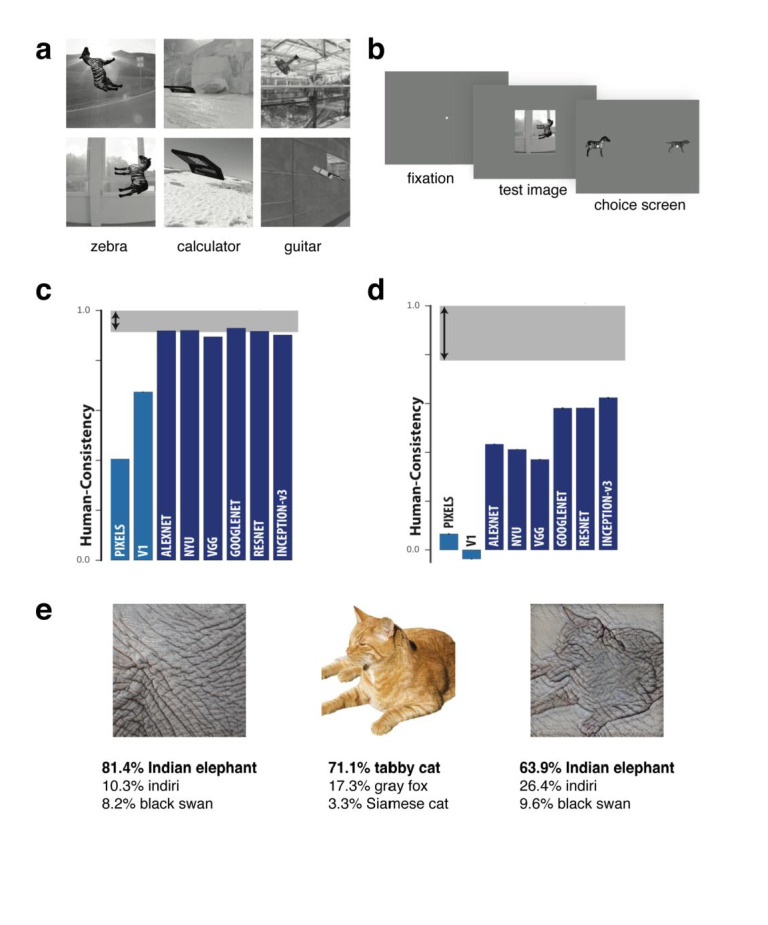
Deep neural networks and object recognition. ( **a**– **b**) Stimuli and behavioral experimental design used in 34. On each trial, human and monkey observers briefly viewed a synthetic test image of a rotated object placed on a random scene background. They then reported which object had been presented by making a binary choice from one of two objects presented in canonical view on the test screen. ( **c**) Results showed that for object category, humans and several different deep neural networks (DNNs) performed similarly. However, humans made different errors than DNNs at the image level ( **d**). ( **e**) Example images from 35. A DNN was more likely to classify an image based on texture (Indian elephant) than shape (tabby cat), whereas human observers do the reverse. Figures a-d were adapted from Rajalingham *et al.* ^34^ under the terms of the Creative Commons Attribution 4.0 International license (CC-BY 4.0).

DNNs have recently achieved human levels of performance in terms of accuracy for image classification ^29^. Specifically, this had been achieved for images from the large database ImageNet and not yet for real world images taken in the wild. An interesting question is to what degree the pattern of successful classification and errors made by DNNs mirror those made by humans making perceptual judgments. Several studies have reported both similarities and differences between human behavior and DNNs. For example, while DNNs can capture human shape sensitivity (with stimuli very different to those on which they were trained) ^31^, they perform less well than simple categorical models in capturing similarity judgements ^32, 33^ and do not capture human sensitivity to properties such as symmetry ^33^. One study that revealed clear differences between human and DNN representations compared the performance of humans, macaque monkeys, and DNNs on an invariant object recognition task ^34^. Stimuli were rendered 3D objects of 24 basic-level categories (e.g. zebra, calculator) superimposed on a natural image background at different orientations/viewpoints ( Figure 1a). Monkey and human subjects viewed these images and then a binary response screen with two objects in canonical view was shown, and their task was to match the object from the previous stimulus ( Figure 1b). Notably, while results for object-level confusion were similar among humans, monkeys, and DNNs ( Figure 1c), performance at the image level did not match between domains ( Figure 1d). This difference in error patterns suggests that accuracy is not an adequate measure of the similarity between humans and DNNs, as vastly different response patterns can yield comparable accuracy.

The observation that humans and DNNs do not show similar patterns of errors at the image level implies that DNNs and humans are not solving the task in the same way or are not relying on the same source of information. A striking demonstration showed that DNNs can be fooled into misclassifying an object by making small changes to the image that are barely perceptible to human observers ^34^. The human visual system is also better able to generalize classification across different forms of noise than DNNs ^36^. An example of clear divergence in the source of information used by humans compared to DNNs is the demonstration that DNNs may favor texture over shape in classifying objects, with the reverse true for human observers ^35^. For example, DNNs such as ResNet-50 trained on ImageNet labelled a picture of a tabby cat rendered with the texture of elephant skin as an "Indian elephant", whereas human observers would label it as "cat" ( Figure 1e). Interestingly, re-training the ResNet-50 architecture to learn a shape-based representation using stylized images in which texture was not predictive of object category led to performance more similar to human observers. Furthermore, there were surprising performance benefits that emerged from the shape-based network, such as greater tolerance to image distortions and better object detection performance.

Beyond comparing network performance with human behavior, recent studies have also compared the representations for objects and scenes within different layers of DNNs to human brain representations measured with fMRI or MEG ^37– 45^. Generally, these studies have found that lower layers of DNNs correlate more with earlier regions within the visual processing hierarchy and higher layers with later regions such as the ventral temporal cortex ^39, 45– 48^. Similarly, time-resolved neuroimaging methods (see also next section) such as MEG have revealed that lower layers of DNNs correlate with human brain activity earlier in time than higher network layers ^37, 40, 47^. However, substantial differences among the human brain, behavior, and DNN representations are also reported, which show that the relationship among them is complex ^38, 39, 41, 44^. For example, for a stimulus set that balanced animacy and appearance, DNNs represented animacy over visual appearance, with the opposite relationship in the ventral temporal cortex ^38^. Similarly, despite striking differences in the representational structure of behavior and fMRI responses, they both showed strong correlations with DNN representations ^39^. Critically, simply calculating correlations is not sufficient for characterizing the similarity between object representations in the human brain and the representations measured by human behavior or in artificial networks. This is because the correlation among these different representations (i.e. among the brain, behavior, and/or DNNs) can be equal in magnitude but explain different parts of the underlying variance. Fundamental progress will be made when we have better methods of revealing what is *driving* the correlation among representations in DNNs, behavior, and the human brain, where such correlations do exist.

There are several emerging directions that may increase the utility of DNNs for advancing our understanding of human object recognition. It is already clear that the link between visual object representations in the brain and DNN representations for the same objects is not straightforward ^38, 39, 41^. Most comparisons have been made with existing pre-trained DNNs; however, deeper insights are likely to emerge from training DNNs to test specific predictions ^35^, which requires systematically varying the task or stimulus set. The addition of biologically plausible architecture to DNNs such as spike-timing-dependent plasticity and latency coding ^49, 50^ may further facilitate the comparison of DNNs and the human brain. For example, the inclusion of recurrent connections more closely captures the dynamic representation of objects in the human brain ^51, 52^. Similarly, transforming the input images to DNNs in a manner similar to the perturbations resulting from the optics of the human eye, for example by applying retinal filters ^34^, may increase the similarity in the underlying representations between these networks and the brain or behavior. One of the most interesting findings thus far has been that DNNs occasionally spontaneously demonstrate features of visual processing that mirror human perception such as generalization over shape or image distortion ^31, 35^. Examination of the conditions under which this occurs may be enlightening for understanding how the human brain achieves object recognition under much more varied viewing conditions and tasks than even state-of-the-art DNNs.

## The temporal dynamics in neural object representations

In recent years, the application of multivariate analyses to time-series neuroimaging methods such as MEG and EEG has facilitated new investigation into the temporal dynamics of cognitive processes. Visual object recognition has been one of the main subfields of cognitive neuroscience to first adapt these methods ^53^. Object recognition is fast ^5^; we can recognize an object in tens of milliseconds. This is much faster than the typical resolution of BOLD fMRI (e.g. 2 seconds); thus, unpacking the temporal evolution of object representations requires alternative neuroimaging methods with millisecond precision. Here we focus on recent work that has revealed the temporal dynamics of object representations in the human brain.

Object representations potentially reflect a number of different properties, which together can be considered to form an “object concept” ^54^. For example, an object concept might include its visual features, the conceptual knowledge associated with an object such as its function, and its relationship to other objects. Neuroimaging methods with high temporal resolution offer the potential to examine the time course of the contribution of these different properties to the underlying object representations. MEG decoding studies have revealed that object identity and category can be decoded in under 100 milliseconds following visual stimulus onset ^18, 19^. The facilitation of objects presented in typical rather than atypical visual field locations occurs around 140 milliseconds ^55^, suggestive of a relatively early contribution of expectation based on visual experience. In contrast, contextual facilitation for classifying the animacy of degraded objects in scenes compared to the same objects presented in the absence of scene context occurs relatively late, 320 milliseconds after stimulus onset, suggestive of a feedback mechanism ^56^.

**Figure 2.**
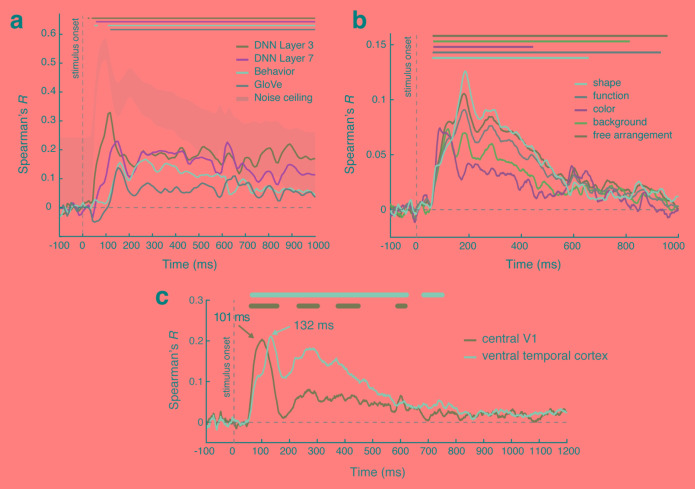
Temporal dynamics of object recognition. ( **a**) The correlation over time between magnetoencephalography (MEG) whole-brain object representations and the representations from several models based on deep neural network (DNN) layers, behavioral similarity judgments, and the Global Vectors for Word Representations (GloVe) model ^37^. Note that the lower DNN layer has an earlier peak than the higher layer. ( **b**) Correlation over time between whole-brain MEG object representations and models based on several different visual and conceptual features ^41^. ( **c**) Functional magnetic resonance imaging–MEG “fusion” reveals a peak correlation between whole-brain MEG object representations and those in the primary visual cortex (V1) at 101 milliseconds (ms) and ventral temporal cortex at 132 ms ^19^. Figure b is adapted from Cichy *et al.* ^41^ under the terms of the Creative Commons Attribution 4.0 International license (CC-BY 4.0).

The contribution of conceptual information to object representations develops after initial visual processing. The emergence of categorical structure based on animacy and real-world object size occurs around 150 milliseconds ^57^. This is consistent with estimates of the lower bound of the formation of conceptual object representations ^37^. Using MEG data recorded for two stimulus sets of 84 object concepts, generalization across exemplars emerged ~150 milliseconds after onset. The shared semantic relationships between the objects was assessed with the Global Vectors for Word Representation (GloVe) model ^58^, an unsupervised algorithm trained on word co-occurrences. Consistent with the time course of generalization around 150 milliseconds, Figure 2a shows that the correlation with the MEG data for behavioral similarity judgements on the stimuli and the GloVe model of semantic information based on word representations both peaked around this time and later than the correlation with representations of the stimuli from an early layer of a DNN. Similarly, the correlation between dynamic MEG representations of objects on their natural backgrounds and measures of behavioral similarity based on shape, color, function, background, or free arrangement is all before 200 milliseconds ^41^ and consists of overlapping representations in time ( Figure 2b). For individual object representations, a model that combines a visual feature model (e.g. HMax ^59^ or AlexNet ^28^ DNN) with a model of semantic features better predicts neural representations measured with MEG than using visual features alone ^60, 61^. The contribution of semantic information to object representations has been linked to activity in the perirhinal cortex ^62^ and anterior temporal cortex ^63^. Collectively, these results are indicative of a relatively early role for conceptual information in object representations that follows the initial visual processing.

Another advantage of studying object representations with high temporal resolution is the potential to disentangle the role of feedforward versus feedback processes in their formation. Feedback is theoretically difficult to study empirically and although its role in visual perception has been acknowledged for decades, the advent of recurrent connections in DNNs ^52^ has reignited interest in attempting to separate the contribution of feedback vs. feedforward processes in object recognition. For example, a computational model incorporating recurrent connections could partially account for occluded object representations measured with MEG, which had a decoding peak much later in time than un-occluded objects ^43^. This suggests feedback processes assist in processing objects under more ambiguous viewing conditions such as occlusion. One recent approach towards isolating the contribution of feedback has been to use the rapid serial visual presentation of objects at very brief presentations under the assumption that rapid presentation disrupts feedback processing of the preceding object(s) ^64, 65^.

One of the challenges the contribution of time-resolved neuroimaging has brought to light is how best to integrate fMRI results with MEG/EEG to elucidate the combined spatial and temporal processing of object recognition. One approach is to use source localization to model the spatial source of the MEG signal in the brain ^52^. An alternative method, fMRI–MEG “fusion”, correlates dissimilarity matrices constructed separately from fMRI and MEG data over time (MEG) and regions of interest (fMRI) ^19, 66^. This approach has been used successfully to demonstrate that whole-brain object representations measured with MEG have a peak correlation earlier in time with the primary visual cortex (V1) and later in time with the ventral temporal cortex ^19, 66^ ( Figure 2c). Furthermore, fusion revealed temporal differences in the contribution of task versus object representations across the visual hierarchy ^67^. Although these results provide a useful validation of the method, the interpretation of fusion results is not straightforward, particularly because of the substantial differences in the spatial resolution between fMRI and MEG. For example, one pair of studies used an object stimulus set that controlled for shape (e.g. snake and rope) across category in order to examine the influence of perceptual and categorical similarity on object representations. Even though the studies used identical stimuli, the results were different between the two neuroimaging modalities: they found more evidence for categorical similarity with fMRI ^68^ and perceptual similarity with MEG ^69^.

The results reviewed above demonstrate the importance of understanding the temporal dynamics of object recognition. So far, multivariate methods applied to MEG and EEG data with high temporal resolution have yielded new insights into the temporal dynamics of semantic versus visual features in object representations and highlighted a possible role for feedback from higher visual areas in the representation of degraded and occluded objects as well as in task-relevant representations. The development of a new generation of wearable MEG systems based on arrays of optically pumped magnetometers promises further advancement in the measurement of brain activity at a high temporal resolution in more varied contexts ^70, 71^. Significant progress will be made with further improvements in linking spatial and temporal neuroimaging data.

## Contextual effects on object representations

Traditionally, object perception has been studied empirically by presenting single objects in isolation on blank backgrounds ^17, 72, 73^. This approach facilitates studying aspects of object recognition such as viewpoint and position invariance without a contribution from the background; however, it likely over-emphasizes the role of object shape. More recently, the context in which object recognition occurs has been increasingly considered in studies aiming to understand the underlying neural mechanisms. This can be the visual context, such as the placement of an object in a scene (either relevant ^39, 56^ or irrelevant ^34^), the action of an agent (e.g. person) involving the object ^74^, or even the use of 3D real objects rather than 2D images ^75, 76^. Or, alternatively, this can be task context, with neural object representations measured as participants perform different tasks on the same object stimuli ^77^. An advantage of all of these approaches with broader scope is that they examine object recognition in circumstances that more closely mimic real-world perception. The results we review here suggest that both visual and task context play a significant role in object processing.

### Visual context: interactions with people and scenes

The simplest form of visual context is to present two objects at a time instead of one. In object-selective cortex, the brain activation patterns to two objects are well-predicted by the average responses to the objects presented in isolation ^78, 79^. More recently, it has been shown that even without the visual context of a detailed scene, the brain representations of objects are affected by expectation driven by context. For example, a fMRI study looked at object pairs taken from scenes (such as a sofa and TV, car and traffic light) presented in their original location versus interchanged locations relative to each other on a blank background ^80^. In the object-selective cortex, the mean of the activation patterns for two isolated objects presented centrally was less similar to the activation patterns for the object pairs when they were in their original location compared with the interchanged location, but this was not the case in the early visual cortex. This suggests that the object-selective cortex is sensitive to the expected location of different objects relative to each other.

**Figure 3.**
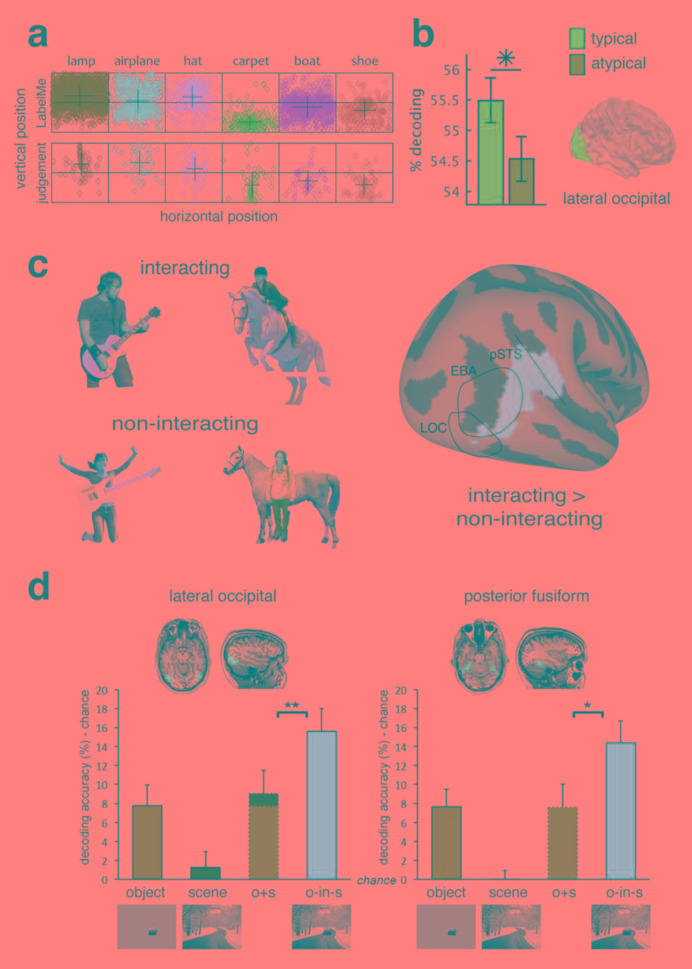
Effect of visual context on the neural representation of objects. ( **a**) Some objects are associated with a typical visual field position in which they tend to occur ^82^. The top row shows object locations from a labelled image database, and the bottom row shows the placement of objects by human observers. ( **b**) In the lateral occipital cortex (LOC), decoding accuracy was higher for objects presented in their typical (e.g. hat in upper visual field) than their atypical (e.g. hat in lower visual field) location. ( **c**) Example stimuli used in 74 of objects in “interacting” and “non-interacting” contexts. A decoding searchlight analysis revealed areas that had higher decoding accuracy for interacting than non-interacting objects. ( **d**) Super-additive decoding accuracy in object-selective lateral occipital and posterior fusiform regions for degraded objects in scenes compared to decoding accuracy for isolated objects or scenes alone ^56^. EBA, extrastriate body area; pSTS, posterior superior temporal sulcus Figure d is adapted from Brandman and Peelan ^56^ under the terms of the Creative Commons Attribution 4.0 International license (CC-BY 4.0).

A related observation is that the location of objects within scenes in the real world is not arbitrary, and objects occur within relatively predictable locations related to their function ^81^. In some cases, this produces a statistical regularity in the visual field location ( Figure 3c). There is some evidence that object processing is facilitated when this expectation is adhered to and objects occur in their typical retinotopic visual field location (i.e. their position relative to the direction of eye gaze). For example, in the object-selective cortex, objects in their typical visual field location (e.g. hat in upper visual field, shoe in lower visual field) could be decoded at a higher rate from the fMRI activation patterns than when they were in the atypical portion of the visual field ( Figure 3d) ^82^. Other higher visual areas in ventral temporal cortex did not show such a difference. EEG results suggest there is a difference in the representation of objects in typical vs. atypical locations as early as 140 milliseconds after stimulus onset ^55^. Overall, the sensitivity of the object-selective cortex to statistical regularities in the location of objects is consistent with the idea of efficient coding in the visual system ^83^, which argues that statistical regularities in the environment can be exploited by neural coding in order to conserve the amount of brain resources engaged in representing the complex visual world.

Another consideration in the representation of multiple objects beyond their relative location is their function. An inherent property of objects is their manipulability, and several studies have investigated how this affects their neural representation ^74, 84, 85^. The degree to which interactions with people and scenes mediates object representations is not homogenous across brain regions. For example, one study examined the effect of interaction on object representations using a stimulus set of humans, guitars, and horses ^74^ ( Figure 3a). They measured brain responses to the isolated objects and for object pairs when they were both interacting (e.g. person riding a horse) and not interacting (e.g. person in front of a horse). In some brain regions, the representation of meaningfully interacting objects was not well predicted by the responses to their individual parts, suggesting coding of the object interaction. For example, a decoding searchlight analysis of the fMRI data revealed areas overlapping with the body-selective extrastriate body area (EBA) and posterior superior temporal sulcus (pSTS) that had higher decoding accuracy for interacting than non-interacting objects ( Figure 3b).

Beyond simple object pairs, similar logic has also been applied to examine how scene context affects object representations. For example, one study measured BOLD activation patterns to degraded objects (either animate or inanimate) presented both in isolation and within intact scenes ^56^. A classifier was trained to distinguish activation patterns associated with animate versus inanimate objects on separate data from intact isolated objects and then tested on the patterns associated with the degraded objects both in isolation and within a scene. In both lateral occipital and posterior fusiform regions, cross decoding accuracy for object animacy was significantly higher for the degraded objects within scenes than that predicted by accuracy for isolated degraded objects and isolated intact scenes ( Figure 3c). However, in scene-selective regions, this was not the case, and decoding accuracy was only additive. These results suggest that object representations in object- but not scene-selective regions are enhanced by the presence of relevant visual context.

Collectively, the studies discussed above highlight the importance of considering the visual context in which objects occur. In the next section, we consider the importance of task context.

### Task context: the stability of object representations in visual cortex

Given that objects are both recognizable and actionable things, an important question is how the neural representation of objects supports behavior. We can make a multitude of judgements about an object, as well as pick them up and use them in action. How do neural object representations change depending on the goal of the observer? In an experimental paradigm, this usually takes the form of keeping the visual stimuli constant and changing the task of the observer. Such changes in task may affect the relevant information and consequently change the distribution of attention. Within the higher visual cortex, where category-selectivity emerges, the majority of results seem to support fairly limited transformation of object representations as a function of task relative to the modulation by object type ^66, 76, 85– 89^. However, in the early visual cortex, there may be strong effects of task, potentially reflecting changes in spatial attention. Consistent with these generalizations, an MEG study found that the impact of task (semantic, e.g. classify the object as small or large, or perceptual, e.g. color discrimination) had a relatively late magnitude effect on object representations across the whole-brain MEG signal rather than a qualitative change to the nature of the representation ^67^. Furthermore, MEG–fMRI fusion suggested the effect of task increased further up the processing hierarchy. Together, this suggests that other brain regions in addition to higher-level visual cortex have an important role in task modulation ^90^. This is in contrast to the effect of visual context reviewed above, in which there was significant modulation of object representations in higher-level visual regions.

Consistent with a locus that is not restricted to the visual cortex, there is considerable evidence for a substantial role of parietal and frontal brain regions in the task modulation of object representations. For example, to address this question, one fMRI study used a stimulus set of 28 objects where semantic category and action associated with the objects were dissociated ^86^. Participants performed two tasks on the same stimuli while within the fMRI scanner: rate objects on a four-point scale from very similar to very different for either hand action similarity or category similarity. For example, pictures of a drum and hammer would be similar for action/manipulation similarity, but drum and violin would be more similar for categorical similarity (both musical instruments). An analysis of the similarity of the brain activation patterns for the different objects revealed that in parietal and prefrontal areas, an action model of the stimuli correlated more with the similarity of object activation patterns during the action task, and vice versa for the category task ^86^. Frontoparietal areas also showed greater within-task correlations than between-task, but this did not differ for occipitotemporal areas. Physical and perceived shape correlated with representations more in occipitotemporal regions. Consistent with this, there is evidence for a difference in the representational space of how objects are represented in occipitotemporal and posterior parietal regions ^91^, with more flexible representations modulated by task in the posterior parietal cortex ^88^.

Collectively, these findings suggest that while task context can affect object representations within the brain, these effects tend to be largest at higher stages of the visual hierarchy with strongest effects in the prefrontal and parietal cortex.

## “Beyond” object recognition

Here we have reviewed three current trends in the field of object recognition: the influence of DNNs, temporal dynamics, and the relevance of different forms of context. These trends have focused the field to consider object representations more broadly rather than object recognition *per se*. Such representations are likely critical for object recognition but will also contribute to many other behaviors ^92^. To conclude, we briefly consider some current challenges in the pursuit of understanding object representations in the human brain and outline some emerging trends that are likely to help push the field forward.

The first issue we consider is what should “count” as an object representation. A consequence of the relevance of visual and task context reviewed in the section above is that it suggests object representations are broader than the particular conjunction of visual properties that visually define the object. The frequent investigation of the neural representation of isolated objects without context may have over-emphasized the role of shape in the underlying representations of real-world objects. Indeed, shape has been found to be a strong predictor of the similarity of the neural representations for different objects ^41^. Similarly, the focus on functional object-selective brain regions, which are localized by contrasts between, for example, isolated objects and scrambled objects ^73^, emphasizes the role of brain regions which are sensitive to shape above other object properties. However, there is evidence that other high-level regions such as scene-selective cortex ^85^ and parietal and prefrontal regions ^86, 88, 90, 91^ are also engaged in object processing. Similar to the importance of visual and task context in object representations, further consideration of object-specific properties such as the role of color ^93, 94^ and material properties ^95^ is likely to provide a new perspective on the nature of object representations. Object representations in the human brain are also tied to other features such as conceptual knowledge ^54^ about their function and relationship to other objects, which are yet to be emulated by DNNs in a way that produces the same flexibility as the human brain.

A second important issue in investigating the nature of object representations is stimulus selection and presentation. In the last decade, there has been concentrated effort to use larger stimulus sets (n = ~100) of objects in neuroimaging event-related designs in an effort to reveal the inherent organization of object categories in brain representations without imposing stimulus groupings in the experimental design ^17^. This is in contrast to blocked stimulus presentation, which is not desirable for investigating representational structure because of inherent biases in the experimental design arising from grouping stimuli together into blocks. However, a limitation of representational similarity analysis ^96^ is that it is relative to the stimulus set used in the experiment and even with ~100 stimuli there are likely to be inherent biases in the stimulus selection. For example, a stimulus set in which shape is a critical difference between stimuli is likely to emphasize a significant role for shape in the organization of the representational space. One recent approach that has potential to move the field forward is the use of very large stimulus sets. Recent databases of 5,000 ^97^ and 26,000 ^98^ visual object images have potential to reveal new insight that has not been possible using experimenter-selected restricted stimulus sets of ~100 images. Additionally, the method used for image selection in the creation of these large stimulus sets is still important in avoiding biases. For example, the THINGS database ^98^ was created by systematically sampling concrete picturable and nameable nouns from American English in order to avoid any explicit or implicit biases in stimulus selection.

Finally, there has been considerable debate over what degree object representations are reducible to the low- and mid-level visual features that co-vary with category membership ^38, 68, 69, 99– 103^. However, this question may be ill-posed. By definition, visual object representations must be characterized by visual features to some degree; even though different object images can be matched for some visual features (e.g. spatial frequency), they will always differ on others (e.g. global form).

In summary, progress in understanding object recognition over the last three years has been characterized by the influence of DNNs, inspection of the time course of neural responses in addition to their spatial organization, and a broader conceptualization of what constitutes an object representation that includes the influence of context. A cohesive understanding of the neural basis of object recognition will also require integrating our knowledge of visual object processing with related processes such as object memory ^104^, which are typically studied independently. Although DNNs have now reached human levels of performance ^28, 29^ for object categorization under controlled conditions, humans perform this task daily under much more varied conditions and constraints. The continued evolution of the field in terms of sophisticated analytic tools, larger stimulus sets, and the consideration of the context in which object recognition occurs will provide further insight into the human brain's remarkable flexibility.

## Footnotes

^a^ We use the term 'object representations' here to mean the measured patterns of response in the brain associated with object perception, rather than a specific internal representation.
